# Fabrication of ulcer-adhesive oral keratin hydrogel for gastric ulcer healing in a rat

**DOI:** 10.1093/rb/rbab008

**Published:** 2021-03-13

**Authors:** Zhongjun Cheng, Rui Qing, Shilei Hao, Yi Ding, Haimeng Yin, GuoDong Zha, Xiaoliang Chen, Jingou Ji, Bochu Wang

**Affiliations:** 1 Key Laboratory of Biorheological Science and Technology, Ministry of Education, College of Bioengineering, Chongqing University, Chongqing 400030, China; 2 School of Chemistry and Chemical Engineering, Chongqing University, Chongqing 401331, China; 3 Bijie Institute of Traditional Chinese Medicine, Bijie City, Guizhou Province 551700, China; 4 Media Lab, Massachusetts Institute of Technology, Cambridge, MA 02142, USA; 5 HEMOS (Chongqing) Bioscience Co., Ltd, Chongqing 402760, China; 6 Department of Nuclear Medicine, Institution of Chongqing Cancer, Chongqing 400030, China

**Keywords:** gastric ulcer, keratin hydrogel, adhesive, oral administration

## Abstract

Hydrogel has been used for in suit gastric ulcer therapy by stopping bleeding, separating from ulcer from gastric fluids and providing extracellular matrix scaffold for tissue regeneration, however, this treatment guided with endoscopic catheter in most cases. Here, we developed an oral keratin hydrogel to accelerate the ulcer healing without endoscopic guidance, which can specially adhere to the ulcer because of the high-viscosity gel formation on the wound surface *in vivo*. Approximately 50% of the ulcer-adhesive keratin hydrogel can resident in ethanol-treated rat stomach within 12 h, while approximately 18% of them maintained in health rat stomach in the same amount of time. Furthermore, Keratin hydrogels accelerated the ethanol-induced gastric ulcer healing by stopping the bleeding, preventing the epithelium cells from gastric acid damage, suppressing inflammation and promoting re-epithelization. The oral administration of keratin hydrogel in gastric ulcer treatment can enhance the patient compliance and reduce the gastroscopy complications. Our research findings reveal a promising biomaterial-based approach for treating gastrointestinal ulcers.

## Introduction

Human all around the world have been suffering from gastric ulcer with a high morbidity and substantial mortality. The formation of gastric ulcer is caused by breaking the balance among defensive factors such as mucus, surface epithelial cells, mucosal barrier, cellular regeneration and aggressive factors including reactive oxygen species, gastric acid and pepsin [1]. The loss of balance is mainly cause by *Helicobacter pylori* infection, stress, smoking, long-term administration of non-steroidal anti-inflammatory drugs (NSAIDs), poor diets and especially alcohol, resulting in mucosal break reaching the submucosa [2–4]. The discovery of effective acid suppressants has greatly decreased the incidence, rates of hospital admissions and mortality of gastric ulcer [5, 6]. However, the management of ulcer disease and relative complications remains a clinical challenge because of the administration of NSAIDs and low-dose aspirin [6, 7].

One of the effective approaches for gastric ulcer healing promotion is the hydrogel therapy [8–12]. Hydrogel coating on the ulcer could stop bleeding and protect the ulcer from gastric fluids, and extracellular matrix (ECM) hydrogel has a direct effect on the wound healing tissue regeneration. In addition, some therapeutic agents can be sustained released from hydrogel to enhance the therapeutic effect [13–15]. However, the traditional hydrogels cannot be directly orally applied to treat gastric ulcer as only a small amount of the hydrogel can contact with ulcer because of the gastrointestinal motility [16, 17], while most of the hydrogel would be expelled from stomach. Therefore, most of hydrogels were assisted with gastroscopy in the gastric ulcer treatment [8–12]. But gastroscopy reduced the patient's compliance, which also carry a risk of several complications, such as internal bleeding, tearing of the lining of oesophagus, stomach or duodenum [18–20].

Keratins have been widely used in wound healing and haemostasis due to their unique characteristics of bioactivity, biocompatibility, biodegradability and natural abundance [21–24]. In our previous studies, keratins are highly promising for wound healing applications, as approximately 90% wound closure was achieved within 10 days after keratin hydrogel treatment [24]. When used for haemostasis *in vivo*, we found that keratins induced high-viscosity gel formation on the wound [25]. We herein speculated that keratin gel could adhesive on the ulcer after oral administration due to its high-viscosity property, and resident in stomach for a long time, which would improve the gastric ulcer healing. Here, the keratins were extracted from human hair and used for hydrogel preparation. Keratin hydrogels with different concentrations were characterized. Ethanol-induced gastric ulcer model in rats was then established to evaluate the ulcer adhesive property and wound healing effect of keratin hydrogels. To the best of our knowledge, this is the first time that keratins were used for peptic ulcer therapy *in vivo*, with specific wound adhesive property.

## Materials and methods

### Materials

Human hairs were collected from local barber shops in Chongqing China. Thioglycolic acid (TGA), sodium dodecyl sulphate (SDS), hydrogen peroxide (H_2_O_2_), hydrochloric acid and sodium hydroxide were purchased From Kelong Chemical Reagent Co., Ltd, China. Malondialdehyde (MDA) kit, Gycogen (PAS) Stain kit and superoxide dismutase (SOD) kit were purchased from the Nanjing Jiancheng Bioengineering Research Institute Co., Ltd. China. Enzyme-linked immunosorbent assay (ELISA) kit was purchased from the Neobioscience Technology Company (Beijing, China). All reagents used in this study are in analytical grade.

### Human hair keratin extraction

The extraction of human hair keratin was conducted according to our previous report [26]. Human hair collected from local barber shops was conductively treated using TGA (0.5 M) under pH 11.0 after washing, which was used to break cystine bonds. The reductive solution was retained, and the crude fraction was extracted in turn by Tris base (100 mM) and deionized (DI) water. Subsequently, the extractions were combined and centrifuged at 6000 rpm for 40 min, and then dialyzed (MWCO 5000 Da) by the ultrafiltration flat sheet membrane (FM1501, Filter & Membrane Technology, Beijing, China). Finally, the pH and of extraction was adjusted to 7.4, and the sample was dialyzed before lyophilization.

### Preparation of keratin hydrogels

Human hair keratin hydrogels (KHGs) were prepared with the concentrations of 10% (KHG1), 15% (KHG2) and 20% (KHG3) (w/v %). Briefly, lyophilized keratins graded into the powders were vortexed in hydrogen peroxide solution (5 wt%) to form a homogeneous dispersion and magnetically stirred drastically in a covered glass container at room temperature. The hydrogels were formed after 3 h stirring. Excessive samples were frozen at 40^○^C and lyophilized by a vacuum freeze drier.

### Morphological observation

The morphology of KHGs was observed with a scanning electron microscope (Nano SEM 400, FEI, Hillsboro, OR, USA) at an accelerating voltage of 20 kV. The KHGs were lyophilized, and a cross-sectional scaffold was placed on an adhesive conducting resin and sputter-coated with the gold for 15 s twice using a Polar on SEM coating system.

### Water absorption studies

A piece of dried KHGs was cut to ∼200 mg pieces and divided into three subgroups randomly. Each group was added into a conical flask filled with 50 ml artificial gastric juice (AGJ). The conical flasks were shaken at temperature of 37°C with 130 rpm. The scaffolds were removed from the AGJ, and the water absorption of KHGs was defined as follows:

$$\mathrm{Water}\;\mathrm{absorption}\;=\frac{Ws-Wo}{Wo}\times 100\%$$

where *W*s and *W*o are weights of fully saturated and dry KHGs, respectively.

### Rheological properties

The rheological properties of KHGs were characterized by a rotational remoter (Gemini HR Nano 200, Malvern, UK) with a diameter 20 mm plate-plate configuration. The oscillatory frequency sweep tests were performed between 0.01 Hz and 10 Hz at a constant strain of 5% at 25°C. The gap between the two plates was set for 0.1 mm. All hydrogels were injected onto the plate through a medicinal ladle. Before the test, hydrogels were degassed by a vacuum pump. The elastic modulus (*G*’) and viscous modulus (*G*”) of the hydrogels were both measured three times.

### Gastric ulcer animal model establishment

All animals experiments were performed following the guidelines from Animal Ethical and Experimental Committee of the Third Military Medical University, China. Sprague–Dawley (SD) rats weighing between 250 g and 300 g were used. They were given water and standard chow, fed with forage and maintained in clean condition kept at temperature of 22 ± 2°C. The SD rats were fasted and given only water for 24 h before implementing a gastric ulcer model. The rats were fed with absolute ethanol (4 ml/kg) through oral gavage and executed for euthanasia to observe the gastric ulcer formed after administering some ethanol for 2 h [27, 28]. The histological changes of stomach such as congestion, oedema, haemorrhage and necrosis were visualized using haematoxylin and eosin (H&E) staining.

### Ulcer-adhesive property evaluation

#### Macroscopic observation

To evaluate the ulcer-adhesive property of KHGs, the ethanol-treated rats and health rats were fed with the KHG2 (1.6 mg/kg) via oral gavage. Their stomachs were then removed under diethyl ether anaesthesia, dissected along the large bay, subsequently washed with 20 ml AGJ so as to wash out the gastric contents, and imaged with a digital camera. In addition, hydrogels on gastric walls were scraped out using a glass slide and imaged. The coverage rate of KHGs on the gastric ulcer was calculated as the area ratio between KHGs and ulcer.

#### Gamma scintigraphy studies

Gamma scintigraphy studies were performed to quantitatively evaluate the amount of KHGs in stomach at different times. The iodine-131(^131^I) was used to label the KHGs by physical absorption [29]. Briefly, keratins were dissolved into 5% H_2_O_2_ solution at different concentrations (100, 150 and 200 mg/ml), and ^131^I solution equivalent to a final radioactivity of 0.4 mci/ml was also added into the keratin solution. The ^131^I-labelled KHGs formed after stirring at 1200 rpm (37°C) for overnight. All procedures were performed in fume hood by a nuclear medicine specialist. The stability of hydrogels labelled with ^131^I was studied.

The ethanol-induced rats weighing about 250–300 g were randomly divided into three groups feeding with ^131^I-labelled KHGs at different concentrations, namely, 100 mg/mL (^131^I—KHG1), 150 mg/ml (^131^I—KHG2) and 200 mg/ml (^131^I—KHG3), respectively. In addition, the healthy rats were also fed with ^131^I—KHG2 as the control group. The KHGs adjusted to the radioactivity of 40uci were administered through a lavage needle after rats were fasted for 24 h. Scintigrams of test preparation were traced via a single-photon emission computed tomography (SPECT) equipment with variable angle dual-detector units (Symbian T2, Siemens, Philadelphia, PA, USA). A region of interest (ROI, a circle with 100 mm diameter) was identified to determine the radioactivity in the stomach at different time points (0 h, 2 h, 4 h, 6 h, 8 h and 12 h). ROI quantification was performed using the acquisition workplace (Siemens Medical Solutions). The radioactivity in the ROI at 0 h was taken as control and designated as 100% [29].

### Ethanol induced ulcer treatments

Ethanol-treated rats were randomly divided into KHG2-treated groups (0.8, 1.6 and 3.2 ml/kg) and vehicle group (1.6 ml/kg of saline). The normal rats were also feed with 1.6 ml/kg of saline as the sham group. All rats were orally administrated by the KHGs or saline once per day, and rats from different groups were executed by euthanasia at 1, 3 and 5 days. Each rat was sacrificed at the homologous moment. The stomachs were removed and opened along great gastric curvature swelling with the normal saline. All stomachs were stored at −80°C.

The gastric wall mucus of each stomach was thoroughly scraped by a hairbrush. The ulcerative lesions were record and graded: 0 = no lesions (normal stomach), 0.5 = hyperaemia, 1 = haemorrhagic spot, 2 = 1–5 small ulcers, 3 = many small ulcers, 4 = 1–5 small and 1–3 large ulcers, 5 = many small and large ulcers; 6 = stomach full of ulcers with some perforations [30, 31]. Each stomach of the whole rats was imaged using a digital camera when the rats were killed without complaints and the wall of stomach was washed cleanly.

### Determination of the ulcer index and the percentage of inhibition

The ulcerative damage area (mm^2^) was statistically determined using image J. The Ulcer Index (UI) for all rats was showed by the ulcerative damage (mm^2^) and the percentage of inhibition (PI %) was calculated according to the following equation [32]:

$$\text{PI \%=}\frac{{\text{GU}}_{C}{\text{ - GU}}_{T}}{{\text{GU}}_{C}{}_{}}\times \text{100 \%}$$

where GU_c_ is the gastric ulcer area of an ulcer model rat and GU_t_ is the gastric ulcer area of a KHGs-treated rat.

### Histopathological evaluation

The stomachs from the rats were fixed in 4% (v/v) formalin solution and stored in the refrigerator, which were then dehydrated with incremental concentration of alcohol and embedded in white paraffin when all alcohol were volatilized. The tissue of the stomach was cut into the piece whose thickness was about 5 μm using a paraffin slicing machine (CUT 4050, MicroTEC, München, Germany). Some of those pieces were stained with haematoxylin–eosin solution (H & E), others were stained with Periodic Acid Schiff (PAS) for analysing the mucosal wall glycoproteins, which were used to evaluate the level of gastric mucosa damage and changes in protonation of the glycoproteins.

### Biochemistry analysis

The tissues stored at −8°C were cut into pieces and weighed to 300 mg for the preparation of a gastric tissue and homogenized using a homogenizer (YHF-DY, Ningbo Scentz Biotechnology, Ningbo, China) in ice-bathed 1 × PBS buffer with 1% cell lysis RIPA. The homogenized solutions were centrifuged at 12 000 rpm for 15 min at 4°C. The supernatant was kept and stored at −80°C for total protein and biochemical factors analysis. The total protein concentration was measured using the BCA protein assay kit (Beyotime Biotechnology, Tokyo, China) according to the manufacturer’s instructions. The activity of SOD, IL-6 and MDA was also determined using commercial assay kits (Jiancheng Bioengineering Institute, Jiangsu, China) following the manufacturer’s instructions.

### Statistical analysis

All measurements were performed at least in triplicate and data were presented as mean ± standard deviation (SD). Tree batches of samples were prepared for each formulation. For selected evaluation tests, the means of all tested formulations were compared with each other by means of a one-way ANOVA with Tukey’s *post hoc* comparisons. The statistical significant level (*P*) was set to be < 0.05.

## Results

### Preparation of the KHGs

The kerateine (reductive keratin) was extracted from human hair using TGA, and disulphide bonds in natural human hair keratin can be broken into thiols [21]. Therefore, the hydrogen peroxide was added into keratin solution to enhance the hydrogel formation. In addition, as the ulcer-adhesive property of keratin hydrogel was due to the increase of hydrogel viscosity, the keratin hydrogels with different concentrations (KHG1 for 10%, KHG2 for 15% and KHG3 for 20%) were fabricated in this study.

**Figure 1.**
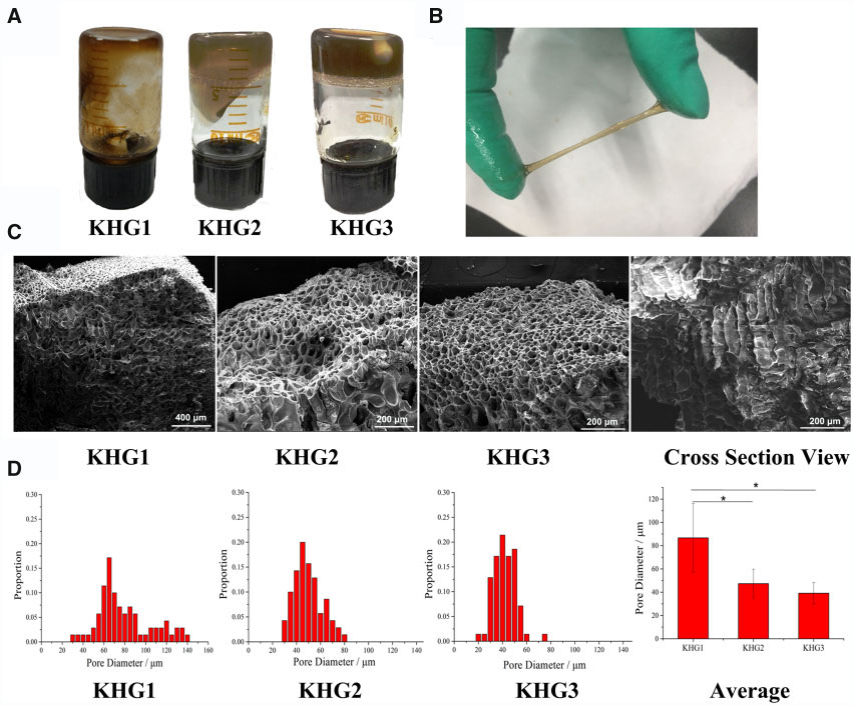
The physicochemical properties of KHGs. (**A**) Photographs of KHGs with different keratin concentrations: 10% (KHG1), 15% (KHG2) and 20% (KHG3). (**B**) Stretching of KHG2. (**C**) SEM images and (**D**) pore diameter distribution of KHGs.

Figure 1a shows the KHGs after an upside-down vertical posture for 5 s. The mobility of KHGs decreased as the concentration increases. In addition, a good malleability of KHG2 can be observed in Fig. 1b.

### Characterization of KHGs

The morphology of KHGs were inspected by SEM. As shown in Fig. 1c, the porous structure of KHGs can be observed. The layer-by-layer structure from the top to the bottom can also be seen on the cross-section, which is resulted from the crosslinking of keratin molecules. In addition, the distribution of pore diameter of KHGs was analysed by statistics analysis using Image J software (Fig. 1d). The average pore diameters of KHG1, KHG2 and KHG3 were ∼39.17 μm, 47.36 μm and 86.73 μm, respectively, which positive correlated to the concentration of KHGs.

**Figure 2.**
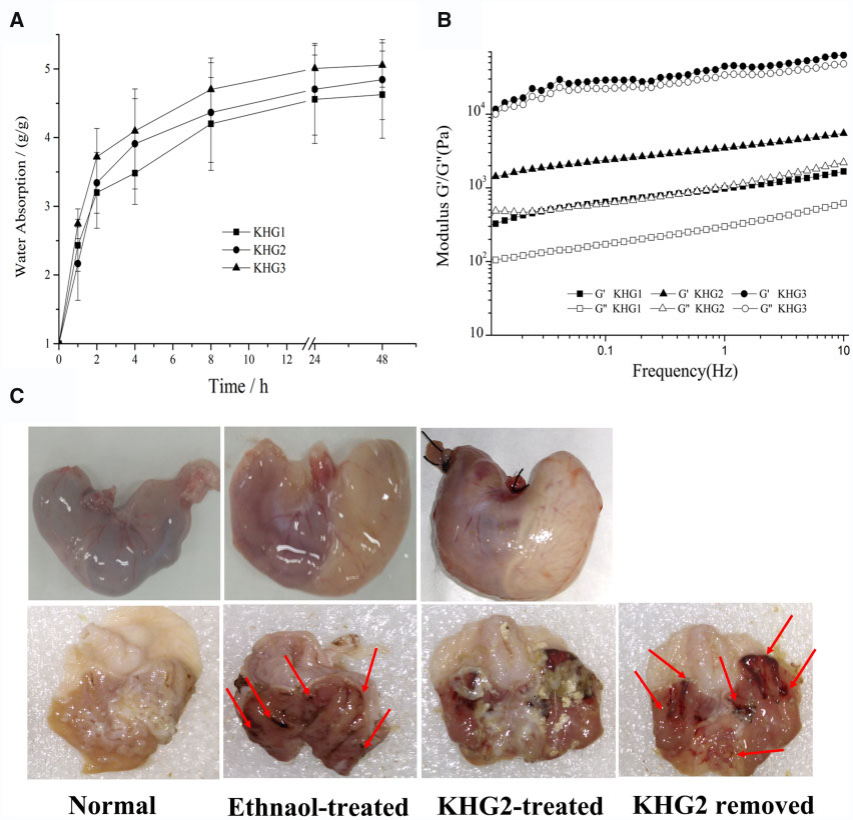
(**A**) Water absorption capacity and (**B**) Elastic moduli (G’) and viscous moduli (G”) versus frequency for the KHGs. (**C**) The macroscopic photos of stomachs of normal rats and ethanol-induced ulcer rats after administration of KHG2 for 2 h. The KHG2 specially adhere to ulcer (red arrows) in the ethanol-induced ulcer rats, and the ulcer can be observed by removing the KHG2 from the stomach.

Water absorption behaviour of KHGs within the stomach is important for its adhesive property and gastric ulcer therapy, which would influence the viscosity of hydrogel, and the gastric fluid absorbed into the KHGs may aggravated the gastric ulcer. Therefore, the water absorption properties of KHGs were investigated and presented in Fig. 2a. All KHGs showed the limited water absorbing capacity in acid condition (pH 1.2). The lyophilized KHG3 swelled four times after incubation 4 h compared to the original KHG3, which increased to five times after 48 h. Besides, the AGJ absorbing capability of KHGs decreased with the decrease of keratin concentration in KHGs, which could be attributed to the different pore sizes and densities at different hydrogel concentrations.

The rheological properties of KHGs were also investigated to determine the linear viscoelastic responses of the hydrogel samples (Fig. 2b). The elastic modulus (G') were greater than the viscous modulus (*G*'') in all three samples within the frequency range analysed, indicating that KHG exhibited the typical behaviour of visco-elastic solids [33]. Furthermore, both the *G*‘ and *G*’' of KHGs increased with the increase in hydrogel concentration. The gel-construct with higher polymer content has higher strength of both elasticity and viscosity.

### Ulcer-adhesive property evaluation

The *in vivo* ulcer-adhesive property of KHGs was directly evaluated using a gastric ulcer rat model, which was established through absolute ethanol oral administration, followed by oral gavage of KHG2. Obvious signs such as haemorrhages mucosal oedema and hyperaemia can be found in the stomach of ethanol-induced rats (Fig. 2c), which indicated that the rat ulcer model was successfully established. In addition, the KHG2 can be observed on the ulcer after 2 h administration, and the ulcer can be found after the KHG2 was swept from the stomach. In parallel, KHG2 was also fed to healthy rat, and no hydrogel was found in the rat stomach at the same time point, illustrating that KHGs has the ulcer adhesive specificity.

**Figure 3.**
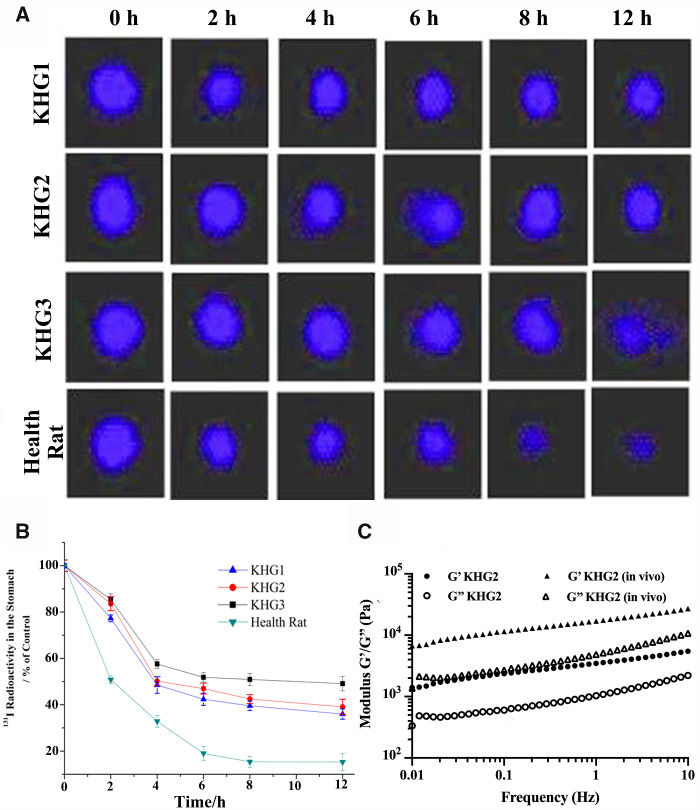
Gastroretentive of KHGs *in vivo*. (**A**) Gammascintigraphic images of ^131^I-labelled KHGs in ethanol-induced ulcer and health rat stomachs. (**B**) Radioactive counts per 2 min of ^131^I-labelled KHGs in the rat stomachs. The values are mean ± SD (*n* = 6). (**C**) Elastic moduli (G’) and viscous moduli (G”) versus frequency for the KHG2 *in vitro* and *in vivo*.

To quantitatively assess the ulcer adhesive property of KHGs, the ^131^I-labelled KHGs adhered to ethanol-induced rat stomach wall were detected using SPECT. The stabilities of ^131^I-labelled KHGs in standard buffer solutions of pH 1.2, 4.5 and 7.4 were evaluated. The activity released from ^131^I-labelled KHGs was less than 8.0% in pH 1.2 and pH 4.5, and less than 6.0% in pH 7.4 within 12 h, respectively. Figure 3a shows the gammascintigraphic images of ^131^I-labelled KHGs in ethanol induced ulcer rats and healthy rats. The radioactivity decreased over time due to the peristalsis of stomach. Furthermore, Fig. 3b displays the radioactive counts per 2 min of ^131^I-labelled KHGs in the stomach within 12 h. Approximately 42% of ^131^I-labelled KHG1, 46% of ^131^I-labelled KHG2 and 51% of ^131^I-labelled KHG3 were retained in the stomach of ethanol induced ulcer rats at 6 h, while only approximately 18% of ^131^I-labelled KHG2 was detected in the stomach of healthy rats. The results indicated that KHGs exhibited a strong ulcer adhesive ability, and the affinity between KHG and ulcer increased with the increase in keratin concentration.

The ulcer adhesiveness of KHGs was attributed the formation of high viscosity of hydrogel on the haemorrhage site. Therefore, we have also detected the rheological properties of KHGs coatings on ulcer. As shown in Fig. 3c, the *G*‘ and *G*’' of KHG2 on the stomach ulcer both observably increased compare to those from native KHG2. The results indicated a rheological property change from KHGs on the haemorrhage site in stomach that can contribute to their ability of ulcer adhesion.

### Ethanol induced ulcer treatment

**Figure 4.**
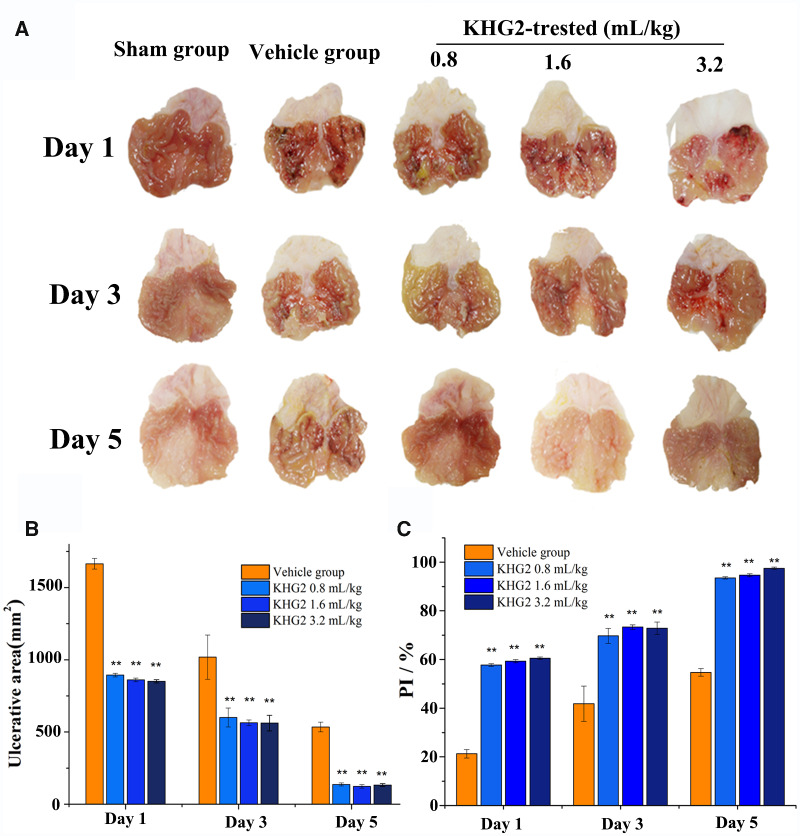
The therapeutic effect of KHGs on ethanol-induced gastric ulcer in rat. (**A**) Macroscopic photograph of stomachs for health rats and ethanol-induced ulcer rats treated with or without different doses of KHG2. (**B**) Effects of different doses of KHG2 on ulcerative area (**C**) and percentage of inhibition (PI %). * *P* < 0.05, ** *P* < 0.01 compared to the vehicle group.

The therapeutic effect of ulcer-adhesive KHGs on gastric ulcer was also evaluated in this study. The stomachs of ethanol induced ulcer rats treated with or without different amounts of KHG2 and healthy rats were removed after killed at Days 1, 3 and 5, which were imaged by a digital camera (Fig. 4a). More obvious ulcer signs, including haemorrhages mucosal oedema and hyperaemia, were found in the stomachs of ethanol induced gastric ulcer rats at Day 1 compare to that in the sham group (healthy rats administrated with saline). In addition, the ulcer area significantly decreased after KHG2 treatment compare to the vehicle group (ulcer rat administrated with saline) (*P* < 0.01) (Fig. 4b), and the ulcer healing in the KHG2-treated rats was also significantly faster than that of the vehicle group at Days 3 and 5 (*P* < 0.01). Meanwhile, the ulcer grade decreased from 5.3 to 0.4 after treated with KHG2 with 3.2 ml/kg within 5 days (Table 1). However, there was no significant difference in ulcer grade between the different doses of KHG2, which indicated that the therapeutic effect of KHGs on gastric ulcer depend on the coverage rate of KHGs on the ulcer, and the excessive KHGs would be expelled from stomach probably due to gastric emptying. Furthermore, the percentage of inhibition (PI) in the KHG2-treated group rose to 97% at 5 days (Fig. 4c), which proved that KHGs facilitated ulcer healing *in vivo*. However, there was no significant difference in wound healing speed between the KHG2-treated groups with different doses, indicating that the amount of KHGs adhered on stomach depended primarily on the ulcer areas, which limited the treatment efficiency.

**Table 1.** The grade distribution of the vehicle group and the KHGs-treated group in rats (mean ± SD, *n *=* *6).

Time	Vehicle group	KHGs-treated group/(ml/kg)		
		0.8	1.6	3.2
Day 1	5.6 ± 0.1	5.3 ± 0.1	5.1 ± 0.2	5.3 ± 0.2
Day 3	4.1 ± 0.1	3.4 ± 0.3	3.4 ± 0.1	3.2 ± 0.4
Day 5	3.2 ± 0.2	0.3 ± 0.3	0.5 ± 0.2	0.4 ± 0.1

### Histopathology of the tissue

**Figure 5.**
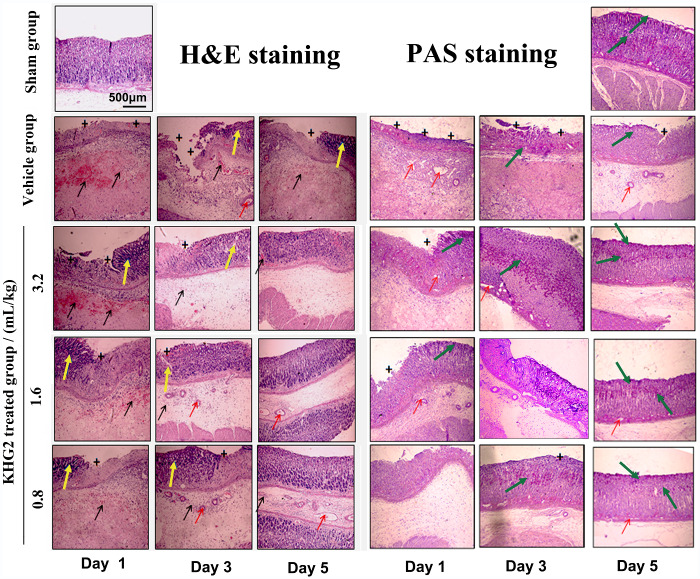
Effect of different doses of KHG2 on histopathology of the stomach tissue (H&E and pas staining, scale bar 500 μm): the plus (+) stands for loss of the stomach glandular structure, the red arrow is the oedema, the yellow arrow represents the disorganization of glandular structure, and the black arrow is on behalf of the haemorrhage for H&E, and the green arrow is the PAS-positive staining (mucosa) for PAS.

The histopathology was then assessed via H&E and PAS straining of the stomach tissue (Fig. 5). The vehicle group displayed some acute gastric tissue injury and disruption of gastric mucosa with severe haemorrhage, submucosal oedema, disorganization of glandular structure and loss of epithelial layer. A smooth gastric mucosa with no stomach glandular structure loss was observed in the sham group. Furthermore, the KHGs-treated groups represented more compact gastric mucosa and less ulcer symptoms compared to the vehicle group, and the ulcer tissues treated with KHG2 can recover from submucosa, muscularis muscle and mucosa within 5 days.

The PAS staining method was used to assess the glycoprotein content in the gastric mucosa. The glycoprotein content of the gastric mucosa significantly decreased in the ulcer rat (vehicle group) compared to the normal rat (sham group) with positive PAS staining. The oral administration of KHG2 with different doses can increase the mucin content, and more positive PAS staining on the top of the gastric mucosa were observed in the KHG2-treated group at Day 3. The result indicted that the KHG could accelerate the regeneration of gastric mucosa to induce a mucus layer.

### Biochemistry factors of the tissue

**Figure 6.**
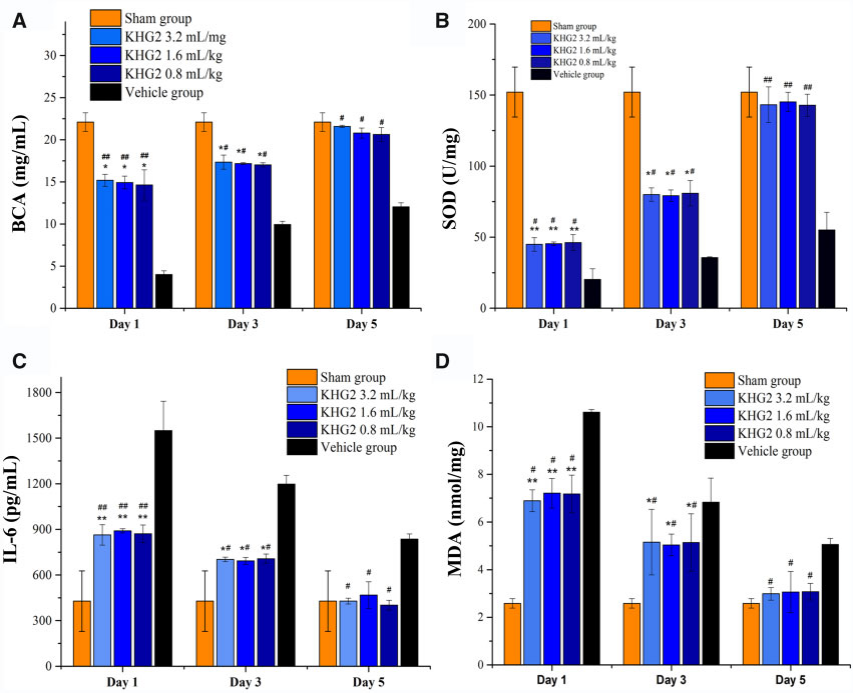
Effect of different doses of KHG2 on the expression of biochemistry factors of the tissue: (**A**) total protein of the homogenatea of the stomach tissue. (**B**) Superoxide dismutase, (**C**) interleukin-6 and (**D**) malondialdehyde of the rat stomachs after different treatments. *P < 0.05, **P < 0.01 compared to the sham group. ^#^*P* < 0.05, ^##^*P* < 0.01 compared to the vehicle group.

The biochemistry factors of the tissue, including superoxide dismutase (SOD), interleukin-6 (IL-6), malondialdehyde activity (MDA) and the total protein in stomach were also detected to evaluate the therapeutic effect of KHGs. The quantity of total protein and SOD in stomachs in different groups was detected and shown in Fig. 6a and b. The SOD and total protein in stomachs significantly decreased in ethanol-treated rats compare to the sham group (*P* < 0.001), and administration of KHG2 markedly up-regulated total protein and SOD levels. There is no significant difference in SOD and total protein levels between KHG2-treated groups and the vehicle group at Day 5. Furthermore, Fig. 6c and d shows the level of IL-6 and MDA in different groups. The IL-6 and MDA levels in stomach significantly increased after the rats treated with ethanol (*P* < 0.01), and the levels of IL-6 and MDA in gastric tissue decreased after KHG2 treatment. Both of them returned to the normal level 5 days after KHG2 treatment. The results indicated that the KHGs can scavenge the oxygen radicals to protect the tissue, mediate the oxidative stress and inhibit the generations of inflammatory cytokines to alleviate the symptoms for the ethanol-induced gastric ulcer.

## Discussion

Polymeric hydrogels have been widely used for wound healing and haemostasis due to their excellent properties in maintaining wettability and moist, keeping the body fluids and providing tissue-like structure. However, hydrogels were usually not used for gastric ulcer treatment because of gastric peristalsis and watery environment [16]. Even though some hydrogels have been applied for gastric ulcer, an endoscopic catheter was usually used for the in suit ulcer therapy [8–12]. While, we developed an oral hydrogel to accelerate the ulcer healing in this study, which can specially adhere to the ulcer without endoscopic guidance. The KHGs can attach to gastric mucosa and ulcer after oral administration, but the hydrogel coating on normal gastric mucosa (healthy rat) can be removed by stomach motility. Additionally, the KHG coatings on the ulcer area can retain in the stomach for a long time (Fig. 2c). After the rat stomachs being dissected after 2 h of KHGs administration, few KHGs were observed in the stomachs of healthy rats. Yet KHGs adhered to the ulcers of ethanol-induced ulcer rats at the same time, while the ulcer can also be found after sweeping the KHGs from stomach. The results indicated that KHGs have the ability of ulcer adhesion on wound for a long time.

The ulcer adhesive mechanism of KHGs was also investigated in this present study. Generally, gastric emptying of foods and drugs can be achieved within 2 h. Some bioadhesive polymers have the potential to prolong the gastric retention time due to their mucoadhesive property. However, bioadhesive particles seem to have the stronger gastretentive ability compare to the hydrogel due to highly specific surface area. We have also studied the bioadhesive property of KHG using healthy rats (sham group in Fig. 3a), and no KHGs were found in the healthy rat stomach after 2 h oral administration. KHGs can be specifically observed on the gastric ulcer at the same time point, which resulted from the formation of high viscosity of hydrogel on the haemorrhage site. The viscosity of KHGs will be significantly increased after mixing with blood (Fig. 3c), which can resist the gastric emptying and cover onto the ulcer specifically for a long time.

Water absorption is another feature of hydrogel, however, the hydrogel used for gastric ulcer treatment should possess limited water absorption ability. Reinforcement of the mucosal barrier and blinding gastric acid can be achieved by the ulcer adhesive coating of KHG, but the ulcer would enmesh into acid environment if the hydrogel can absorb a mass of stomach acid. Thus, the hydrogel instead of freeze-dried KHGs was orally administrated to treat gastric ulcer in the present study. The KHGs showed limited water absorbing capacity in the acidic condition, and the lyophilized KHG3 swelled to five times within 48 h, which indicated that KHG3 (200 mg/ml of keratin hydrogel) would absorb few gastric fluids in the stomach.

Malleability of hydrogel is needed for the therapy of gastric ulcer because of the stomach motility and distention. The stomach wall will motor regularly during the gastric emptying. Normal capacity of human stomach ranges from 0.25 to ∼1.7 l influenced by the position of the body, the condition of surrounding viscera and organs, the amount and type of food and the digestion time [34]. Therefore, sufficient rheological properties of KHGs are necessary to resist stomach motility and expansion. As-prepared KHGs showed good malleability *in vitro* (Fig. 1b), while the rheological properties of KHGs were enhanced *in vivo* because of the formation of high-viscosity gel.

Apart from the ulcer adhesive property, keratin was chosen to treat gastric ulcer in the present study on account of their strong wound healing capability. Keratins, derived from human hair, feather and wool, have been used for the wound repair [22, 24]. Thus, pure keratins without drugs or growth factors were employed to accelerate the gastric ulcer. The ulcer grade decreased from 5.3 to 0.3 after treated with 0.8 ml/mg of KHG2 within 5 days, and the ulcer tissues can recover from submucosa, muscularis muscle and mucosa. The results proved keratins’ strong gastric wound healing ability. However, no significant difference in ulcer grade between the different doses of KHG2 was noted, indicating that the therapeutic effect of KHGs on gastric ulcer depended primarily on the coverage rate of KHG on the ulcer, and the excessive KHGs would be expelled from stomach due to gastric emptying.

Besides the effect of ulcer healing, the KHGs also displayed the therapeutic effect on gastric bleeding in this study. Actually, the excellent haemostatic property of keratin has been reported in the previous study [25, 35]. While keratin was used for gastric bleeding treatment in the present study for the first time. The haemostasis can be found when KHGs were swept from the gastric ulcer (Fig. 2c), which was explained by the fact that high-viscosity gel can form when keratin mixed with blood, and then supply a pressure effect to stop bleeding. The rebleeding may occur when the sealing function was broken. Although the gastric ulcer animal model can be successfully established by traditional methods, the animal models of ulcerative bleeding are difficult to control. Therefore, the bleeding time and mass of blood loss in gastric ulcer rate with and without the KHGs treatment were not assessed in this study.

Keratin extracted from human hair has been used for haemostasis, wound healing, drug delivery and cell culture in the previous studies [21, 25, 36], and the biocompatibility of human hair keratin implanted in subcutaneous and injected into the brain has been investigated. The results showed that no significant histopathological differences of the major organs (brain, heart, lung, spleen, kidney and liver) were observed between the control and keratin groups, and the volume of keratin decreased over time, and most of keratin hydrogels degraded *in vivo* within 28 days after intracerebral infusion and subcutaneous implantation [24, 37, 38].

Furthermore, highly adhesive hydrogels have been successfully designed for tissue regeneration based on the adsorption, mechanical interlocking theory, diffusion and electrostatic interactions [39]. The typical mussel-mimetic hydrogels possess ultrahigh adhesion energy on wet biological tissues with defined cross-linkers achieved via catechol dimerization [40, 41], and the bioadhesive supramolecular gelatin hydrogels fabricated via the host–guest complexation between the aromatic residues of gelatin and free diffusing photo-crosslinkable acrylated β-cyclodextrin monomers [42]. Furthermore, a strong adhesive hydrogel with high H-bond density formed after incorporation of ‘triple hydrogen bonding clusters’ into matrix through a unique ‘load sharing’ effect without any chemical reaction [43]. While, the KHG displayed a strong adhesion on ulcer surface due to the high-viscosity gel formation, and a novel strategy to adhesive hydrogel design supplied in the present study.

## Conclusion

In summary, keratin, for the first time, was used for the gastric ulcer treatment in this study. Compared to the hydrogels used in previous studies, keratin hydrogels can be orally administrated directly without endoscopic guidance because of its ulcer-adhesive property, which can adhere to the gastric ulcer due to the formation of high viscosity gel, and resist the gastric emptying more than 12 h. Furthermore, KHGs accelerated the gastric ulcer healing via two ways. On the one hand, a layer of compact barrier was formed after the KHGs coating on the wound, which can stop the bleeding and prevent the epithelium cells from gastric acid damage. On the other hand, KHGs alleviated the symptoms of the gastric ethanol-induced ulcer including the bleeding, oedema, ulcer with macroscopy and abnormal expression of IL-6, MDA and SOD. It is predictable that the developed KHG will have a significant impact on the clinical gastric ulcer treatment in the future.

## Funding

The authors acknowledge the financial assistance provided by the National Natural Science Foundation of China (11972099 and 31600770), the Venture & Innovation Support Program for Chongqing Overseas Returnees (cx2020079), the Visiting Scholar Foundation of Key Laboratory of Biorheological Science and Technology (Chongqing University), Ministry of Education (CQKLBST-2017-008), the research and development talent base subject of advantageous traditional Chinese medicine in Bijie City, Guizhou Province (RCJD2020-21) and the projects in the National Science & Technology Pillar Program during the Twelfth Five-year Plan Period (2015BAI05B03).

*Conflict of interest statement*. None declared.
